# Characterizing the Bounce and Separation Dynamics of Janus Drop on Macrotextured Surface

**DOI:** 10.3390/polym14122322

**Published:** 2022-06-08

**Authors:** WooSeok Choi, Sungchan Yun

**Affiliations:** Department of Mechanical Engineering, Korea National University of Transportation, Chungju 27469, Korea; w.choi@ut.ac.kr

**Keywords:** Janus drop, macrotextured surface, interfacial dynamics

## Abstract

Janus drops are thermodynamically stable when a high-viscosity fluid is imposed on a low-viscosity fluid. To understand physical mechanisms in Janus drop impact on macrotextured surfaces, several challenges in finding parameters or strategies still remain. Here, this study investigates the asymmetric bounce and separation of impinging Janus drops on non-wettable surfaces decorated with a macroridge to explore the effect of the drop size, viscosity ratio, and ridge size on the dynamics. Through numerical simulations, we determine the threshold Weber number, above which separation occurs, by varying drop diameters and viscosity ratios of the Janus drops. We investigate the initial bouncing directions of separated drops as a function of the impact velocity and viscosity ratio. We also predict how the separation efficiency is affected by the ridge’s height and width. The asymmetric impact dynamics of Janus drops on macrotextured surfaces can provide new strategies to control drop bouncing in applications, such as liquid separation and purification.

## 1. Introduction

Compound drop manipulation is of fundamental and practical interest in diverse fields, including drug delivery [1], combustion [2], 3D printing [3], and self-cleaning [4,5,6]. The impingement of the compound drop, such as Janus, core–shell, and particle-laden configurations, can bring more opportunities for applications owing to their particularities and numerous combinations of complex drops and complex surfaces [7,8]. Extensive studies have been carried out to investigate the mechanisms of the drop bouncing and the methods for bouncing promotion or suppression [9,10]. The drop dynamics can be governed by the theoretical Rayleigh limit given by

*τ_o_* = (*ρD*_0_^3^/8*σ*)^1/2^, (1)

where *ρ*, *D*_0_, and *σ* are the density of the liquid, equilibrium diameter, and interfacial tension, respectively [11,12]. There are relevant dimensionless numbers:

*We* = *ρD*_0_*U*_0_^2^/*σ*, (2)

*Oh* = *μ*/(*ρD*_0_*σ*)^1/2^, (3)

where *U*_0_ is the impact velocity and *μ* is the viscosity of the liquid. Here, Weber number (*We*) indicates the inertia relative to the surface forces, and Ohnesorge number (*Oh*) represents the viscous relative to the inertial and surface forces.

Recently, Yu et al. characterized the spreading and splashing behavior of Janus drop impacts through experiments [13]. They demonstrated that Janus drops were thermodynamically stable when a high-viscosity fluid was imposed on a low-viscosity fluid. The length of diffusion between the fluids was too small compared to the size of the drop, allowing the drop to act as a two-fluidic system. They also investigated the splashing threshold of the low-viscosity component decreased with an increasing viscosity of the high-viscosity component. Blanken et al. [14] observed that the water-in-oil emulsion droplet could rebound on the hydrophobic and hydrophilic surfaces owing to a self-lubricating layer between the core drop and the wetting surface. Damak et al. [15] found an emulsion drop’s transition to bouncing–sticking–bouncing with the help of a formation of the liquid-impregnated surface as the impact velocity increased.

Many researchers have focused on an exploration of the basic physical principle and practical meaning as related to liquid–solid interaction, including drop impact on micro- and nano-structured surfaces [16,17,18,19,20,21,22,23,24,25,26,27,28]. Bird et al. [16] studied that superhydrophobic surfaces with macrotextures could decrease the contact time of millimeter-sized water drops by means of center-assisted retraction. The macrotexture played a role in redistributing the mass and momentum. Gauthier et al. [17] observed a steplike reduction in residence time on repellent wires with increasing impact velocities. They investigated the impact dynamics by varying drop sizes, wire sizes, and impact velocities. Besides the head-on impact on macrotexture, Han et al. [18] presented fin–stripe non-wettable surfaces for spatial offset maximization and temporal contact minimization of bouncing drops. Other methods have been developed to functionalize the surfaces and modify the performance: vitrimer thin films for durable hydrophobicity [19], functionalized copper surfaces [20], and biphilic surfaces [21] for enhanced heat transfer.

Meanwhile, several efforts have been made to study viscous drop impact on macrotextured surfaces. Abolghasemibizaki et al. [22] demonstrated steplike reduction in the residence time for viscous drop impact on non-wettable surfaces with a single wire. They also found that the residence time was independent of impact velocity for the inertial-capillary regime, whereas it increased with impact velocity for the viscous-capillary regime. Raiyan et al. [23] studied the role of viscosity in the impact dynamics on the superamphiphobic surface with macrotextures for varying impact velocities. Our group found that the initial shape of the impinging drop could alter the impact behavior and shorten both the bounce height and residence time significantly when elliptical footprint drops collided on heated [24], flat non-wettable [25], and ridged non-wettable surfaces [26,27]. The role of viscosity of impinging drops in bouncing dynamics was studied numerically by investigating the residence time on single-ridge surfaces [27]. Most recently, our group demonstrated the possibility of the separation of the low-viscosity component from the high-viscosity component on macroridge structures by reducing the residence time [28]. The previous study predicted the bounce and separation behavior for various Weber numbers, viscosity ratios, and initial inclined angles with respect to the ridgeline. However, to further understand physical mechanisms in Janus drop impact on macrotextured surfaces and find strategies in practical terms, fundamentals of how the dynamics of the Janus drop are affected by the sizes of drop and ridge and viscosity ratio should be provided.

**Figure 1:**
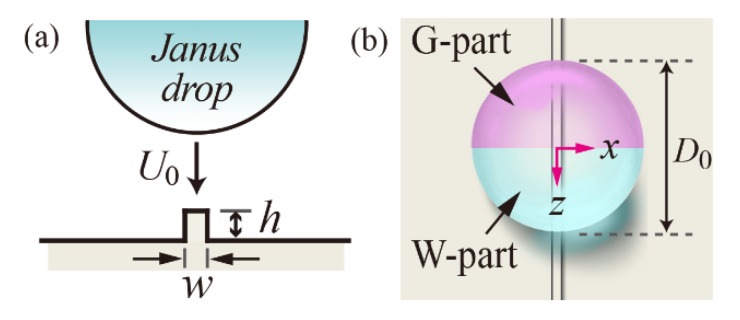
(**a**) Schematic of an impinging Janus drop upon non-wettable solid surfaces decorated with a rectangular ridge at the initial diameter *D*_0_ and impact velocity *U*_0_. The basic size of the ridge is controlled to the height of *h/D*_0_ = 0.2 and width of *w/D*_0_ = 0.05. (**b**) Janus drops contain two phases of W- (water) and G-parts (glycerin/water mixture) that share a common interface perpendicular to the solid surface.

In the study, we investigated the asymmetric bounce and separation of impinging Janus drops on non-wettable surfaces decorated with a macroridge to explore the effect of the drop size, viscosity ratio, and ridge size on the dynamics. By attaching a high-viscosity component into a low-viscosity component, Janus drops consisted of two fluidic parts: one was a W-part named after water; the other was a G-part named after glycerin/water mixture, as shown in Figure 1. We assumed the impact configuration in which the interface between the two fluidic parts was normal to the solid surface. Through numerical simulations, we determined the threshold Weber number of the W-part (*We_c,w_*), above which separation occurred, and the residence time of the W-part (*t_c,w_*) by varying drop diameters and viscosity ratios of the Janus drops in the volume of fluid (VOF) method [29]. The initial bouncing directions of separated drops were investigated as a function of the impact velocity and viscosity ratio. Lastly, we predicted how the separation efficiency was affected by the heights and widths of ridges. The findings can provide strategies to control drop bouncing in applications, such as liquid purification [30,31] and additive manufacturing [32,33].

## 2. Numerical Methods

The VOF method was employed to predict the interface of the Janus drops on a macrotextured surface. The VOF algorithm reported by Rider and Kothe [34] was used to trace the interface. Water (W-part) and glycerin/water mixture (G-part) were used as two phases of Janus drops, surrounded by air at atmospheric pressure and room temperature as a third phase. The local volume fraction *ψ_i_* for each phase *i* can be represented as $\;\overset{3}{\underset{i=1}{\sum }}\psi_{i}=1$. Numerical schemes were based on previous methods for the prediction of liquid–solid interaction [25,35]. The three-dimensional mass and momentum equations were calculated in the domain as follows:

(4) $$\frac{\partial }{\partial t}\left(\rho \right)+\nabla \cdot \left(\rho \vec{v}\right)=0$$

(5) $$\frac{\partial }{\partial t}\left(\rho \vec{v}\right)+\nabla \cdot \left(\rho \vec{v}\vec{v}\right)=-\nabla p+\rho \vec{g}+\nabla \cdot \left[\mu \left(\nabla \vec{v}+{\left(\nabla \vec{v}\right)}^{T}\right)\right]+\sigma \kappa \nabla \psi_{i},$$

where density and viscosity are represented as $\rho =\overset{3}{\underset{i=1}{\sum }}\psi_{i}\rho_{i}$ and $\mu =\overset{3}{\underset{i=1}{\sum }}\psi_{i}\mu_{i}$, respectively, *κ* is the curvature of the interface $\kappa =-\left(\nabla \cdot \vec{n}\right)$, $\vec{n}$ is the unit vector normal to the interface, and $\vec{g}$ is the gravitational acceleration. As the simplest model, the continuous interfacial force approximation was employed for the calculation of interfacial tension in the last term of the momentum equation [36]. The advection of the volume fraction was applied in the model as $\partial \psi_{i}/\partial t+\nabla \cdot (\psi_{i}\vec{v})=0$. The temporal and spatial derivatives were discretized by utilizing the first-order implicit scheme and convective models reported by Leonard [37], respectively. We made a rectangular computational domain of 11 × 11 × 6 mm^3^ in size and a mesh resolution of at least 55 cells per *D*_0_. We also densified the cells near bottom surfaces self-adaptively to improve the precision of computation. For the velocity and pressure fields, the normalized residuals were set to be smaller than 10^–5^. The maximal internal iteration and minimal time step were chosen as 40 per time step and 0.1 μs, respectively.

The basic size of the ridge was controlled to the height of *h/D*_0_ = 0.2 and width of *w/D*_0_ = 0.05. The ridge’s size varied under the ranges of *h/D*_0_ = 0.05 − 0.5 and *w/D*_0_ = 0.025 − 0.4. In the domain, the initial spherical shapes containing W- and G-parts with a certain *D*_0_ were patched near the macroridge. Table 1 presents the liquid properties for two fluidic parts in a certain weight percentage, measured by [22]. A static contact angle model was employed to characterize the wettability of the ridge and surface in the current work. The current study set the contact angle of 160° as the wetting condition because of dynamic angles (~160°) for the advancing and receding and too small contact angle hysteresis (<4°), reported by [22].

Several viscosity ratios between W- and G-parts (*α ≡ μ_g_*/*μ_w_*) are represented as *α*_1_ − *α*_4_, as shown in Table 1. The subscripts of physical quantities, *w* and *g*, indicate the properties of W- and G-parts, respectively. W-parts have the equilibrium diameter *D_w_* = *D*_0_/2^1/3^ derived from volume conservation and impact velocity *U*_0_ = 0.5 − 2.5 m/s. For W-parts, the interplay between the inertial and surface forces is important, and the viscous force is negligible because of *Oh_w_* = 0.003 at *D*_0_ = 2.0 mm. For G-parts, the viscous force can govern the impact dynamics because *Oh_g_* = 0.099 − 4.22 at *D*_0_ = 2.0 mm. The *t_c_* is defined as a residence time on the surface. Ω represents the separated volume relative to the initial volume for W-parts, called as a separation efficiency. The axial momentum *p* is calculated from the volume integral of the axial momentum divided by the initial momentum.

To validate the current simulation, we compared the numerical data of maximum spreading diameters (*D_m_*) with the fitting lines of experimental data obtained from single-phase viscous drop impact on flat surfaces [22]. We introduced a dimensionless number, called impact number (*P = We/Re*^4/5^) to estimate whether capillarity or viscosity ruled the hydrodynamics. Clanet et al. [12] suggested the relations of *D_m_/D*_0_ *~ We*^1/4^ for *P* < 1 and *~ Re*^1/5^ for *P* > 1. Afterward, the experimental demonstration presented the numerical coefficient of 0.83 in the relations as follows [22]:

*D_m_/D*_0_ = 0.83 *We*^1/4^ for *P* < 1, (6)

*D_m_/D*_0_ = 0.83 *Re*^1/5^ for *P* > 1. (7)

**Figure 2:**
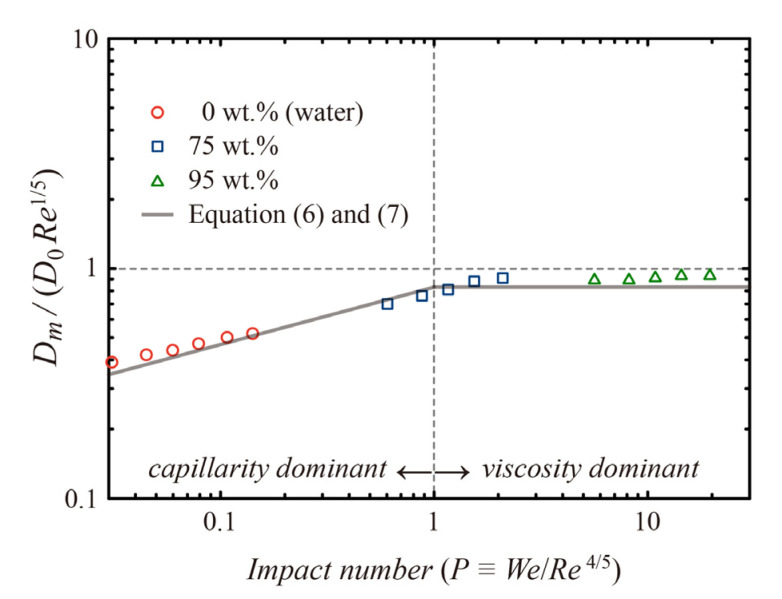
Model validation based on the maximum spreading diameter obtained from single-phase drop impact on non-wettable flat surfaces.

Our numerical data of water and glycerin/water mixtures (75 and 95 wt.%) agree with Equations (6) and (7), as shown in Figure 2. When the impact number passes unity, it can be a transition point from capillarity to the viscosity dominance in the drop dynamics.

## 3. Results and Discussion

**Figure 3:**
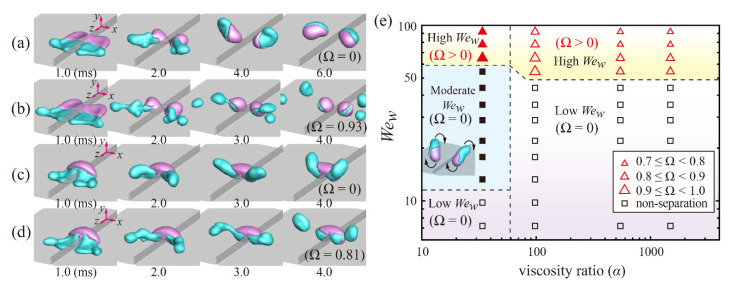
Bouncing characteristics depending on the impact velocity and viscosity ratio at *D*_0_ = 1.33 mm. (**a**–**d**) Morphological behavior of the impinging drops at (**a**,**b**) *We_w_* = 44 and 65 under the viscosity ratio of *α*_1_, respectively, and (**c**,**d**) *We_w_* = 44 and 65 under the viscosity ratio of *α*_3_, respectively. (**e**) Regime map of *We_w_* as a function of the viscosity ratio. Triangle and square symbols in the map indicate separation/non-separation situations between W- and G-parts, respectively; filled and open symbols represent fragmentation/non-fragmentation situations for G-part, respectively. The Weber numbers can be characterized into three regimes based on the separation between the two parts and the fragmentation of G-part: low-*We_w_* (W-part still adhering to G-part), moderate-*We_w_* (symmetric division of G-part by the ridge, but Ω = 0), and high-*We_w_* regimes (W-part separated from G-part).

Bouncing characteristics of drops on the macroridge structures can depend on the drop size, impact velocity, and viscosity ratio. Figure 3a–d show the morphological behavior of the impinging drops under the different impact velocities and viscosity ratios at *D*_0_ = 1.33 mm. In the low-viscosity ratio (*α*_1_), W- and G-parts exhibit large deformations throughout the spreading and retraction, as depicted in Figure 3a,b. After touching the surface at smaller *We_w_*, the W-part spreads more widely in the *x*-direction than the *z*-direction, as shown in Figure 3a at 1.0 ms. The film of the part shrinks toward the G-part, thereby leading to symmetric division and bounce of the two parts, as illustrated at 6.0 ms. Note that the G-part is redistributed into two symmetric parts only for *α*_1_. For a high *We_w_*, the film of the W-part is thinner and wider compared to that observed at the smaller *We_w_*, and thus the newly formed inner rim strongly retracts toward the outer rim along the *x*-direction, as seen in Figure 3b at 2.0 ms. A large portion of the W-part is lifted off from the surface and then separated from the G-part (i.e., Ω = 0.93). Distinct from the dynamics in the low-viscosity ratio, the notable difference is observed between the dynamics of W- and G-parts in the high-viscosity ratio (*α*_3_). The W-part is still attached to the G-part at a low *We_w_* (Figure 3c), whereas it is partly split from the G-part at a high *We_w_* (Figure 3d). A higher inertia of the W-part allows the drop to be separated from the G-part (i.e., Ω = 0.81), compared to the dynamics in the low *We_w_*.

For a quantitative understanding, the bounce and separation behavior can be further investigated by introducing a regime map as a function of *We_w_* and *α*, as illustrated in Figure 3e. Triangle and square symbols in the map indicate separation/non-separation situations between W- and G-parts, respectively. Filled and open symbols represent fragmentation/non-fragmentation situations for the G-part, respectively. The *We_w_* can be characterized into three regimes based on the separation between the two parts and the fragmentation of the G-part: low-*We_w_* (W-part still adhering to G-part), moderate-*We_w_* (symmetric division of G-part by the ridge, but Ω = 0), and high-*We_w_* regimes (W-part separated from G-part). The transitions of the regimes can be recognized at *We_w_* = 65 and 54 for *α*_1_ and *α*_3_, respectively. Approximately 80% of the W-part or more (Ω ≥ 0.8) can be separated from the G-part at the *We_w_* thresholds. In other words, the higher the viscosity ratio, the easier it is for the W-part to separate from the G-part. It is found that the *We_w_* threshold for separation at *D*_0_ = 1.33 mm is slightly higher than that observed at *D*_0_ = 2.0 mm (*We_c,w_* ~ 40) [28]. In addition, the volume ratio of the separated W-part decreases slightly with increasing *We_w_* and *α*, which can be seen by the triangle symbol’s size described in Figure 3e. The highly viscous G-part shrinks slowly, pinned on the surfaces, which can act as a resistance to the splitting of the W-part.

**Figure 4:**
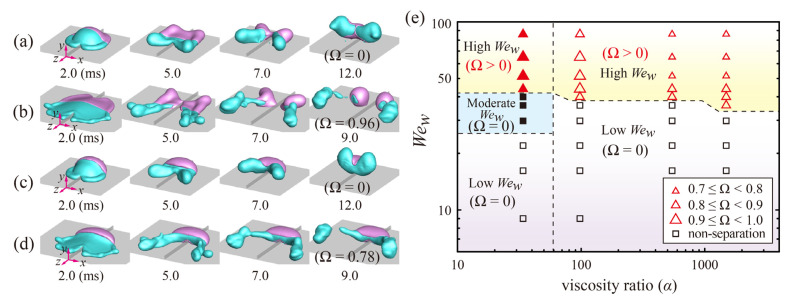
Bouncing characteristics depending on the impact velocity and viscosity ratio at *D*_0_ = 3.0 mm. (**a**–**d**) Shape evolutions of the drops impinging at (**a**,**b**) *We_w_* = 22 and 65 under the viscosity ratio of *α*_1_, respectively, and (**c**,**d**) *We_w_* = 22 and 65 under the viscosity ratio of *α*_3_, respectively. (**e**) Regime map of *We_w_* as a function of the viscosity ratio. Triangle and square symbols in the map indicate separation/non-separation situations between W- and G-parts, respectively; filled and open symbols represent fragmentation/non-fragmentation situations for G-part, respectively. The Weber numbers can be characterized into low-, moderate-, and high-*We_w_* regimes for W-part.

To further examine the role of the drop size in Janus drop impact, we also investigated the bouncing behavior of larger drops (*D*_0_ = 3.0 mm) in Figure 4. In a low-*We_w_* regime, the W-parts still adhere to G-parts throughout the impact, and the entire drops bounce off from the macroridge surface for any *α*, as shown in Figure 4a,c. In a high-*We_w_* regime, a large portion of the W-part leaves the surface and detaches from the entire drop with Ω = 0.96 and 0.78 for the viscosity ratios of *α*_1_ and *α*_3_, as depicted in Figure 4b,d, respectively. In a regime map, the transitions can be found at *We_w_* = 44 and 40 for *α*_1_ and *α*_3_, respectively. Approximately 80% of the W-part or more (Ω ≥ 0.8) can be separated from the G-part at the *We_w_* thresholds. It is found that the *We_w_* threshold for separation at *D*_0_ = 3.0 mm is almost similar to that observed at *D*_0_ = 2.0 mm (*We_c,w_* ~ 40) [28]. It is found that Ω decreases slightly with increasing *We_w_* and *α*, which can be seen by the triangle symbol’s size described in Figure 4e. It is also found that the range of the moderate *We_w_* for high *D*_0_ is much narrower than that observed at low *D*_0_.

**Figure 5:**
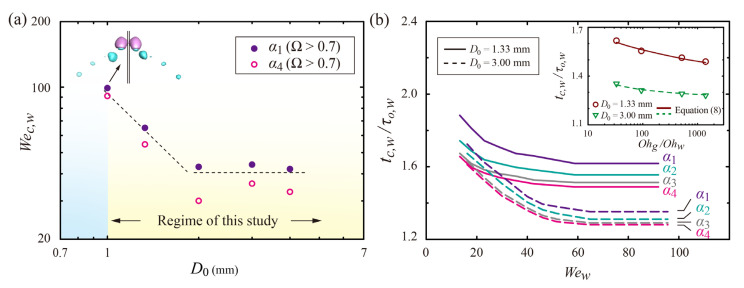
(**a**) *We_w_* thresholds for the separation under different drop sizes. Filled and open symbols represent the thresholds under the viscosity ratios of *α*_1_ and *α*_4_, respectively. (**b**) Residence time of W-parts normalized by *τ_o,w_* as a function of *We_w_* at two distinct drop diameters. The inset represents the normalized residence time as a function of *Oh_g_*/*Oh_w_*, obtained from numerical data (symbols) and predictions from Equation (8) (lines) under the high-*We_w_* regime. The data regarding *D*_0_ = 2.0 mm in (**a**) are reprinted with permission from [28]. Copyright 2022 AIP Publishing.

Figure 5a shows the *We_w_* thresholds for the separation under different drop diameters. Filled and open symbols represent the thresholds under viscosity ratios of *α*_1_ and *α*_4_, respectively. The figure reveals that the *We_w_* threshold increases with decreasing *D*_0_. It is found that a smaller drop diameter (*D*_0_ < 1.0 mm) leads to low efficiency for the separation (i.e., Ω < 0.3). Since the drop size is much smaller than the capillary length (~(*σ*/*ρ*g)^1/2^), the capillary force can be significantly dominant, and the W-part can be hardly separated from the G-part. To improve the separation efficiency of the smaller drops, splashing would be helpful because the high inertia may break the W-part into small drops by attaining dominance over the capillary and viscosity forces. For larger *D*_0_, the drops maintain the low-*We_w_* thresholds, compared to the small *D*_0_. The large drop size can induce the relatively high inertia of the bouncing W-part and the relatively long residence time of the G-part, thereby resulting in the high efficiency of the separation at the low *We_w_*.

Figure 5b shows the residence time of W-parts normalized by *τ_o,w_* as a function of *We_w_*, where the inertial-capillary timescale is given as *τ_o,w_ =* (*ρD_w_*^3^/8*σ*)^1/2^. The graph reveals that *t_c,w_/τ_o,w_* decreases significantly in low- and moderate-*We_w_* regimes and has only slight variations in the high-*We_w_* regime for any *α*. The inset represents the normalized residence time as a function of *Oh_g_*/*Oh_w_*, obtained from numerical data (symbols) and predictions (lines) under the high-*We_w_* regime. To explain the trend of the decreasing *t_c,w_/τ_o,w_*, we employed a theoretical model related to a receding velocity of the film and conservation of mass from the previous study [16,28]. The residence time can be determined by durations for spreading and retraction as ${\tilde{t}}_{c}$ = ${\tilde{t}}_{s}$ + ${\tilde{t}}_{r}$, where the dimensionless time $\tilde{t}$ is denoted as $\tilde{t}$ = t/*τ_o,w_*. Here, the retraction time can be categorized by the retraction behavior of outer and newly formed inner rims as ${\tilde{t}}_{r}$ = ${\tilde{t}}_{r1}$ + ${\tilde{t}}_{r2}$ + ${\tilde{t}}_{r3}$, where ${\tilde{t}}_{r1}$ is the time required for reaching a maximal spread of the outer rims, ${\tilde{t}}_{r2}$ is the time required for the collapse between the outer and newly formed rims, and ${\tilde{t}}_{r3}$ is the time required for the oscillation until the drop bounces off the surface. The maximal spread in the *x*-direction (*x_m_*) can be written as *V_f_* ${\tilde{t}}_{r1}$*τ_o,w_* + 2*V_f_* (${\tilde{t}}_{r}-{\tilde{t}}_{r1}$ − ${\tilde{t}}_{r3}$)*τ_o,w_*, where *V_f_* is the average receding velocities of the outer and inner rims, defined by *V_f_* = (2*σ*/*ρh_f_*)^1/2^, where *h_f_* is the film thickness [38]. In addition, the mass conservation yields that (1/6)π*ρD_w_*^3^ ~ π*ρx_m_*^2^*h_f_*. Therefore, the residence time can be scaled by incorporating the previous expressions as

(8) $${\tilde{t}}_{c}={\tilde{t}}_{s}+{\tilde{t}}_{r}={\tilde{t}}_{s}+(1/2){\tilde{t}}_{r1}+{\tilde{t}}_{r3}+6^{-1/2},$$

where ${\tilde{t}}_{r1}$ ~ 0.95(*Oh_g_*/*Oh_w_*)^–1/5^ and ~ 0.82(*Oh_g_*/*Oh_w_*)^–1/2^ that are fitted in a high-*We_w_* regime (*We_w_* > 60), obtained from the simulation for *D*_0_ = 1.33 and 3.0 mm, respectively. In addition, ${\tilde{t}}_{r3}$ can be set constant because the deformed W-part is released within a timescale of *τ_o,w_* during the oscillation. Equation (8) can reproduce numerical results once ${\tilde{t}}_{r3}$ is set to 0.35, as shown in the inset of Figure 5b. Moreover, the equation can confirm the trend that ${\tilde{t}}_{r}$ obtained in a high-*We_w_* regime relies on the viscosity ratio, regardless of *We_w_*.

**Figure 6:**
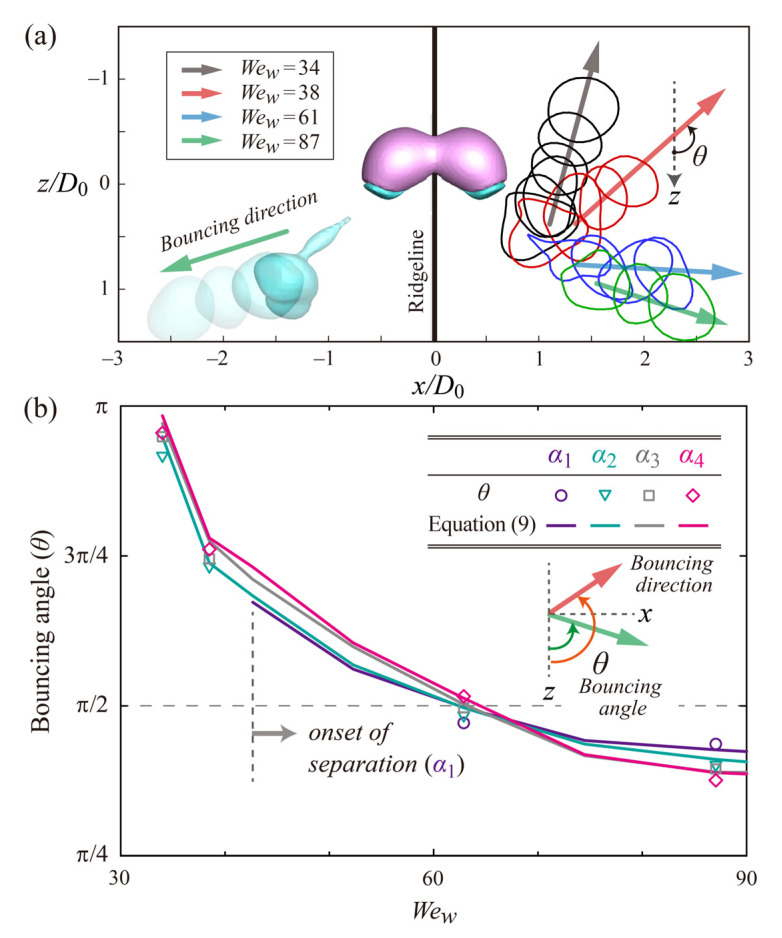
Initial bouncing directions of separated drops as a function of *We_w_* and viscosity ratio. (**a**) Sequential images of top-view snapshots in intervals of *Δt* = 2*D*_0_/*U*_0_ for the estimation of bouncing directions under different *We_w_* in the case of the viscosity ratio of *α*_2_. (**b**) Bouncing angle with respect to *+z*-direction as a function of *We_w_* for four distinct viscosity ratios. Symbols and lines represent *θ* measured from trajectories and estimated from the characteristic momentum ratios between the horizontal directions during retraction, *p_z,ret_*/*p_x,ret_*, respectively.

The initial bouncing directions of separated drops can depend on the impact velocity and viscosity ratio, as shown in Figure 6a. To estimate the bouncing direction, we obtained sequential images of the top-view snapshots for different *We_w_* at the viscosity ratio of *α*_2_. The right column of the figure indicates that separated drops present the velocity components of *−z*-direction at low *We_w_*, whereas they present the velocity components of *+z*-direction at high *We_w_*. For example, there is the strongest velocity component of *−z*-direction at the *We_w_* thresholds (i.e., *We_c,w_* = 34). The findings can be explained by the residence time depending on the impact velocity. After spreading, W-parts that still adhere to G-parts retract in *−z*-direction at low *We_w_*. In this situation, the retraction time of the W-part is significant in increasing residence time. However, W-parts are being separated from G-parts during spreading at high *We_w_*. In this situation, the velocity components of *+z*-direction can be dominant in the bouncing dynamics. We investigated bouncing angles with respect to *+z*-direction as a function of *We_w_* for four distinct viscosity ratios, as shown in Figure 6b. The bouncing angle decreases approximately from π to 3π/8 as *We_w_* increases approximately from 30 to 90, but it is almost independent of the viscosity ratio. Previous studies showed that the behavior of bouncing drops in the retracting process could be characterized by a constant momentum for a certain principle direction and that the momentum ratios were related to the initial direction of bouncing [26]. We plotted in Figure 6b the momentum ratios between the horizontal directions by introducing an expression as

*θ* = π/2 − tan^−1^(*p_z,ret_/p_x,ret_*), (9)

where *p_z,ret_* and *p_x,ret_* represent the characteristic constant momenta in *z*- and *x*-directions, respectively. The data from Equation (9) are in good agreement with those measured from the drop’s trajectories. The findings of the bouncing directions can provide a post-processing strategy to control drop bouncing in applications, such as liquid separation.

**Figure 7:**
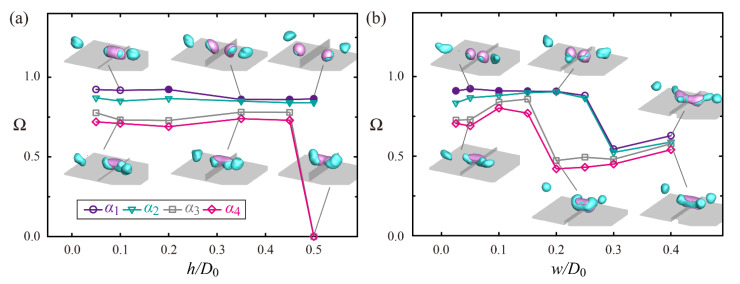
Dependency of Ω on (**a**) height and (**b**) width of the ridge. Filled and open symbols represent the fragmentation/non-fragmentation situations for G-part, respectively. Snapshots of the insets are obtained from drop impact on macroridges with (**a**) *h*/*D*_0_ = 0.1, 0.35, and 0.5 and (**b**) *w*/*D*_0_ = 0.05, 0.2, and 0.4 for two viscosity ratios of *α*_1_ and *α*_3_. The Weber number in (**a**,**b**) corresponds to *We_w_* = 61.

**Figure 8:**
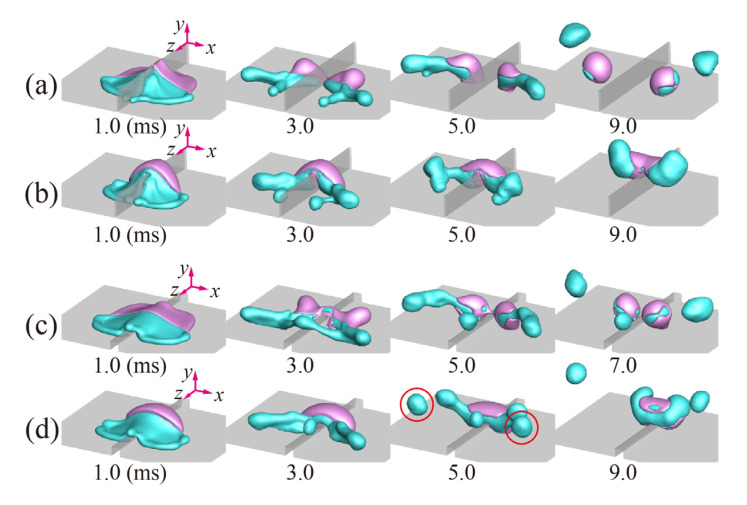
Shape evolutions of the drops with the viscosity ratios of (**a**,**b**) *α*_1_ and *α*_3_ under *h*/*D*_0_ = 0.5 and *w*/*D*_0_ = 0.05, respectively, and (**c**,**d**) *α*_1_ and *α*_3_ under *h*/*D*_0_ = 0.2 and *w*/*D*_0_ = 0.2, respectively. The Weber number in (**a**–**d**) corresponds to *We_w_* = 61.

We investigated the effect of the height and width of the ridge on the separation efficiency under *h/D*_0_ = 0.05 − 0.5 and *w/D*_0_ = 0.025 − 0.4. Figure 7a shows that approximately 70% or more of W-parts can be separated from G-parts at *h/D*_0_ < 0.5 and that the low-viscosity ratio gives rise to the high Ω for any *h/D*_0_. The bouncing dynamics on the tallest ridge of *h/D*_0_ = 0.5 are shown in Figure 8a,b. Drops with *α*_1_ exhibit the normal separation mechanisms belonging to the high-*We_w_* regime, whereas drops with *α*_3_ are not split because climbing of the G-part along the ridge causes weak relative velocities between two fluidic parts, as seen in the figures at 5.0–9.0 ms. Meanwhile, it is found in Figure 7b that approximately 70% or more of W-parts can be separated from G-parts at *w/D*_0_ < 0.2. The bouncing dynamics on the ridge with *w/D*_0_ = 0.2 are shown in Figure 8c,d. The W-parts of the drops with *α*_1_ are split into two main separated drops on the surface and a small drop remaining on the ridge, as seen in Figure 8c at 5.0–7.0 ms. However, drops with *α*_3_ eject daughter drops at the end of W-parts (red circle), and the remaining W-parts with the reduced inertia retract toward G-parts, thereby leading to low Ω as depicted in Figure 8d at 5.0–9.0 ms. Note that the high inertia of W-parts and long residence time of G-parts can be necessary to attain the high efficiency of the separation. The geometrical effect of the ridge on Ω can be stated as follows: the separation efficiency of Ω ≥ 0.7 can be secured with *h/D*_0_ = 0.25 ± 0.2 and *w/D*_0_ = 0.1 ± 0.05.

As an additional note, we studied the effect of the drop size, ridge size, and offset distance on the dynamics of Janus drops with an initial inclination of 0° against the ridgeline. For high heights and low widths of ridges, the W-part can be separated from the G-part with high efficiencies of the separation under the impact configurations. Details are described in Appendix A.

## 4. Conclusions

We studied the bouncing characteristics of Janus drops for various drop sizes, viscosity ratios, and ridge sizes by using the VOF method. The Janus drop contained the two phases of W- (water) and G-parts (glycerin/water mixture) that shared a common interface perpendicular to solid surfaces. Bouncing and breakup behavior was characterized by regime maps that could be divided into several regimes based on the separation between the two fluidic parts and the fragmentation of G-part: low-*We_w_* (W-part still adhering to G-part), moderate-*We_w_* (symmetric divisions of two fluidic parts), and high-*We_w_* regimes (W-part separated from G-part with high Ω). We found that the range of the moderate *We_w_* became wider with decreasing *D*_0_ and that the *We_w_* thresholds for the separation increased with decreasing *D*_0_. In general, the higher the viscosity ratio, the easier it was for W-parts to separate from G-parts. The initial bouncing directions of separated drops depended on the impact velocity and viscosity ratio. The bouncing angle decreased approximately from π to 3π/8 as *We_w_* increased approximately from 30 to 90, but it was almost independent of the viscosity ratio. The bouncing angles estimated from momentum ratios between the horizontal directions were in good agreement with those measured from the drop’s trajectories. Predictions of separation efficiency at different *h/D*_0_ and *w/D*_0_ showed that the high inertia of W-parts and long residence time of G-parts could be necessary to secure the high efficiency of the separation. We suggest that the threshold *We_w_*, above which separation occurs, can be regulated by adjusting the drop diameters and viscosity ratios of the Janus drops.

## Figures and Tables

**Table 1:** Liquid properties of two fluidics parts. Reprinted with permission from [22]. Copyright 2019 American Chemical Society. Reprinted with permission from [28]. Copyright 2022 AIP Publishing.

Part	Liquid	*ρ* (kg/m^3^)	*μ* (Pa s)	*σ* (N/m)	Viscosity Ratio (*α* *≡* *μ_g_/μ_w_*) (−)	*Oh_g_/Oh_w_* (−)
W-part	Water	998.2	0.001	0.072	-	-
G-part	Glycerin/water (75 wt.%)	1185	0.0339	0.064	33.9 (*α*_1_)	33.0
	Glycerin/water (85 wt.%)	1210	0.0977	0.064	97.7 (*α*_2_)	94.1
	Glycerin/water (95 wt.%)	1248	0.545	0.064	545 (*α*_3_)	516
	Glycerin (100 wt.%)	1261	1.491	0.064	1491 (*α*_4_)	1406

**Figure A1:**
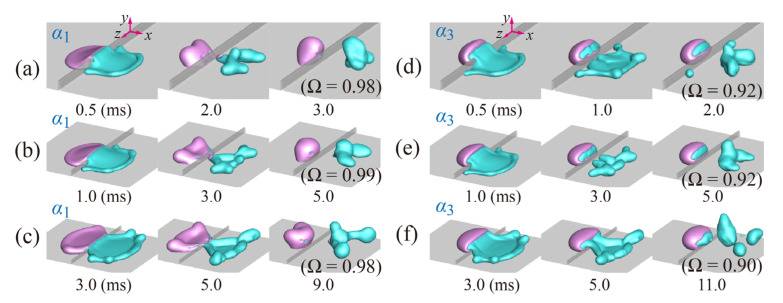
Shape evolutions of the drops with (**a**–**c**) *D*_0_ = 1.33, 2.0, and 3.0 mm under the viscosity ratios of *α*_1_, respectively, and (**d**–**f**) *D*_0_ = 1.33, 2.0, and 3.0 mm under the viscosity ratios of *α*_3_, respectively. The sizes of the ridges shown in (**a**–**f**) are controlled to the height of *h/D*_0_ = 0.2 and width of *w/D*_0_ = 0.05. The Weber number in (**a**–**f**) corresponds to *We_w_* = 61.

**Figure A2:**
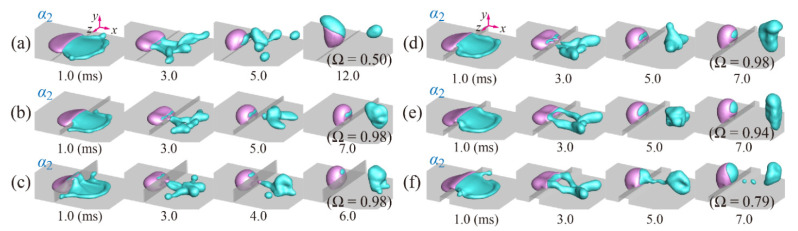
Shape evolutions of the drops on the ridged surfaces with (**a**–**c**) *h*/*D*_0_ = 0.05, 0.2, and 0.5 and (**d**–**f**) *w*/*D*_0_ = 0.1, 0.2, and 0.4 under the viscosity ratio of *α*_2_, respectively. The basic size of the ridge corresponds to *h/D*_0_ = 0.2 and *w/D*_0_ = 0.05. The drop’s diameter and Weber number in (**a**–**f**) correspond to *D*_0_ = 2.0 mm and *We_w_* = 61.

**Figure A3:**
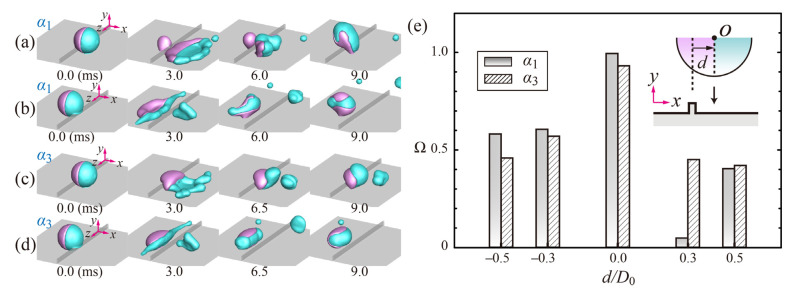
Off-centered Janus drop impact on the surfaces at *We_w_* = 61. Morphological behavior of the impinging drops at (**a**,**b**) *d/D*_0_ = +0.3 and –0.3 under the viscosity ratio of *α*_1_, respectively, and (**c**,**d**) *d/D*_0_ = +0.3 and –0.3 under the viscosity ratio of *α*_3_, respectively. (**e**) The ratio Ω as a function of *d/D*_0_ under the viscosity ratios of *α*_1_ and *α*_3_.

## Data Availability

The data presented in this study are available on request from the corresponding author.

## Appendix A

This appendix deals with the study on the effect of the drop size, ridge size, and offset distance on the dynamics of Janus drops with an initial inclination of 0° against the ridgeline.

Figure A1 shows the shape evolutions and separations for varying drop sizes under the viscosity ratios of *α*_1_ and *α*_3_. After touching the surfaces, the W- and G-parts differently behave between the ridges throughout the impact. The W-parts are split by ridges into approximately equal sizes (i.e., Ω ≥ 0.9) for any *α*. In striking contrast to W-parts exhibiting the large deformation, the highly viscous G-part shrinks slowly and hardly deforms when a liquid bridge between two fluidic parts is generated, thereby leading to the relatively low efficiency of the separation, as shown in Figure A1d–f.

We investigated the effect of the height and width of the ridge on the separation efficiency under *h/D*_0_ = 0.05 − 0.5 and *w/D*_0_ = 0.1 − 0.4 at an initial inclination of 0°, as shown in Figure A2. It is found that, for high heights and low widths of ridges, the W-parts can be separated from the G-parts with high efficiencies of the separation (i.e., Ω > 0.9). For the shortest ridge, the thin film of the W-part shrinks more slowly along the ridge compared to that on other heights of ridges, which can cause the low efficiency of the separation (i.e., Ω = 0.5), as depicted in Figure A2a. It is known that the retraction velocity of the film on the ridge can increase when the drop impacts on a tall macroridge, following a relation of *V_p_* ~ (2*σ*/[*ρ*(*h_f_* − *h*)])^1/2^ [16], where *V_p_* is the velocity on the ridge and *h_f_* is the film thickness. For the widest ridge, the relatively thick film remaining on the ridge can suppress the fast retraction along the ridge for the detachment of the W-part, which can result in the relatively low efficiency of the separation (i.e., Ω = 0.79), as seen in Figure A2f.

Additionally, we predicted the off-centered impact on the macroridge surfaces at an initial inclination of 0°. Figure A3a–d show the morphological behavior of the drop at the off-centered distance *d/D*_0_ = +0.3 or –0.3. After touching the surface at *α*_1_, the G-part is redistributed into two parts between the ridge, and the W-part is still attached to the G-part (i.e., Ω ~ 0), as depicted in Figure A3a. For *d/D*_0_ = –0.3 at the same viscosity ratio, the W-part is split into unequal sizes by a ridge with Ω ~ 0.6 and spreads widely in the *z*-direction at the left side of the ridge, as seen in Figure A3b. When the viscosity ratio increases, the G-part is no longer split, and thus the residence time of the part is relatively longer. Accordingly, a portion of the W-part is lifted off from the surface and then separated from the G-part with Ω ~ 0.45, as illustrated in Figure A3c. The W-part that is off-centered at *d/D*_0_ = –0.3 is separated from the G-part with Ω ~ 0.6, as shown in Figure A3d. We plotted in Figure A3e the volume ratio of the separated W-part as a function of *d/D*_0_ for the viscosity ratios of *α*_1_ and *α*_3_. In general, the Ω obtained for each viscosity ratio is slightly higher at a negative *d/D*_0_ (separation owing to the breakup by a ridge) than a positive *d/D*_0_ (separation owing to asymmetric bouncing without a ridge).
